# Reactivity of [Pt(P^*t*^Bu_3_)_2_] with Zinc(I/II) Compounds: Bimetallic Adducts, Zn–Zn Bond Cleavage, and Cooperative Reactivity

**DOI:** 10.1021/acs.organomet.1c00088

**Published:** 2021-04-13

**Authors:** Nereida Hidalgo, Carlos Romero-Pérez, Celia Maya, Israel Fernández, Jesús Campos

**Affiliations:** † Instituto de Investigaciones Químicas (IIQ), Departamento de Química Inorgánica and Centro de Innovación en Química Avanzada (ORFEO−CINQA), Consejo Superior de Investigaciones Científicas (CSIC) and University of Sevilla, Avenida Américo Vespucio 49, 41092 Sevilla, Spain; ‡ Departamento de Química Orgánica I and Centro de Innovación en Química Avanzada (ORFEO−CINQA), Facultad de Ciencias Químicas, Universidad Complutense de Madrid, Madrid 28040, Spain

## Abstract

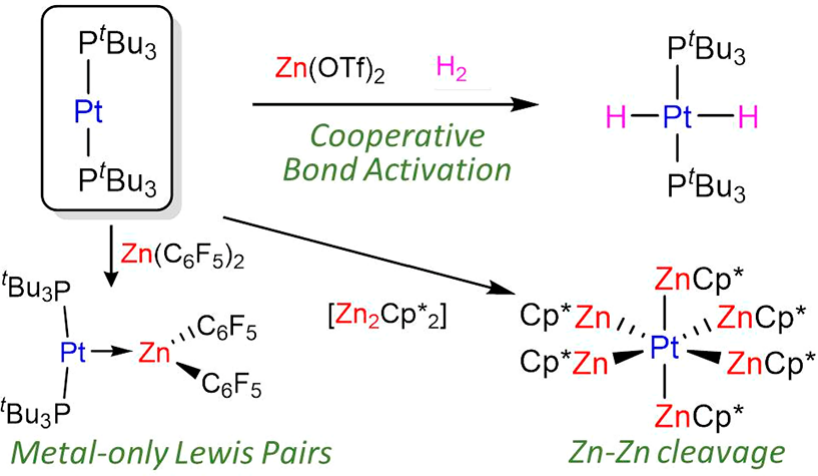

Metal-only Lewis pairs (MOLPs) based on zinc electrophiles are particularly interesting due to their relevance to Negishi cross-coupling reactions. Zinc-based ligands in bimetallic complexes also render unique reactivity to the transition metals at which they are bound. Here we explore the use of sterically hindered [Pt(P^*t*^Bu_3_)_2_] (**1**) to access Pt/Zn bimetallic complexes. Compounds [(P^*t*^Bu_3_)_2_Pt → Zn(C_6_F_5_)_2_] (**2**) and [Pt(ZnCp*)_6_] (**3**) (Cp* = pentamethylcyclopentadienyl) were isolated by reactions with Zn(C_6_F_5_)_2_ and [Zn_2_Cp*_2_], respectively. We also disclose the cooperative reactivity of **1**/ZnX_2_ pairs (X = Cl, Br, I, and OTf) toward water and dihydrogen, which can be understood in terms of bimetallic frustration.

## Introduction

The unique features of bimetallic complexes are behind the rapid development that has recently taken place in the field.^1^ Among these complexes, metal-only Lewis pairs (MOLPs),^2^ that is, bimetallic compounds in which the two metal atoms are held together exclusively by a dative M → M bond, constitute a fascinating family. MOLPs constructed around Lewis acidic zinc(II) fragments are particularly appealing due to their relevance to Negishi cross-coupling catalysis. In fact, intermediates containing dative Pd → Zn interactions are crucial to accessing low-energy transition states during transmetalation^3^ and play essential roles in *cis*/*trans* isomerization^4^ and deleterious homocoupling processes.^5^ Bergman and Tilley have shown that biaryl reductive elimination from Pt(II) compounds is accelerated upon Zn(C_6_F_5_)_2_ coordination,^6^ while Whittlesey and Macgregor have explored a heterobimetallic Ru/Zn compound and demonstrated that the unsaturated “ZnMe” terminus promotes C–H reductive elimination and dihydrogen activation at the Ru(II) site.^7^ Although Zn-based MOLPs remain rare^8^ these findings highlight the opportunities that may emerge from combining zinc electrophiles with electron-rich transition metal compounds.

As for Lewis basic fragments, [Pt(PCy_3_)_2_] (Cy = cyclohexyl) is likely the most extensively investigated donor in MOLP chemistry.^9^ In fact, recently reported [(PCy_3_)_2_Pt → ZnBr_2_] is the first well-defined unsupported M → Zn(II) adduct.^10^ Combination of the bulkier analogue [Pt(P^*t*^Bu_3_)_2_] (**1**) with a Cu(I) species revealed bimetallic cooperation during O–H bond activation.^11^ Similarly, we have explored the reactivity of [(P^*t*^Bu_3_)_2_Pt → AgNTf_2_] (NTf_2_ = bis(trifluoromethanesulfonyl)imide), which readily cleaves X–H (X = H, C, O, and N) bonds across the Pt–Ag linkage, while the parent monometallic components remain inactive.^12^ On these grounds, we decided to inspect the formation and reactivity of zinc-containing MOLPs based on [Pt(P^*t*^Bu_3_)_2_] (**1**). In doing so, we have examined its reactivity with a range of zinc precursors, more precisely ZnX_2_ (X = Cl, Br, I, and OTf; OTf = trifluoromethanesulfonate), ZnR_2_ (R = Me, Et, Ph, η^5^-C_5_Me_5_, and C_6_F_5_), and the more exotic Zn(I) dimer [Zn_2_(η^5^-C_5_Me_5_)_2_] (Zn_2_Cp*_2_).^13^

## Results and Discussion

**Scheme 1:**
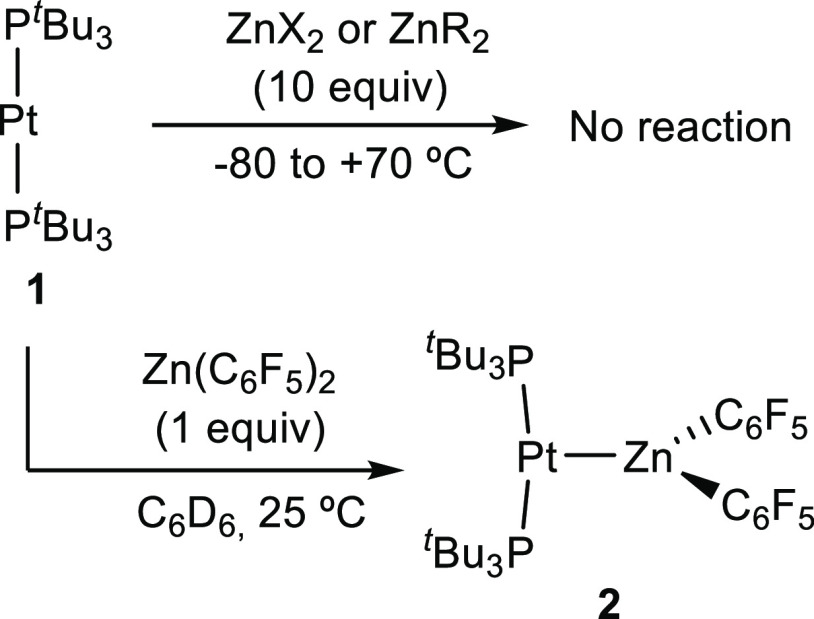
Reaction of [Pt(P^*t*^Bu_3_)_2_] (**1**) with Zinc (Pseudo)halides and Organozinc Compounds Top: X = Cl, Br, I, and OTf; R = Me, Et, and Ph, C_5_Me_5_; solvent = C_6_D_6_, CD_2_Cl_2_, THF, or C_6_H_5_F.

Treatment of [Pt(P^*t*^Bu_3_)_2_] (**1**) with zinc (pseudo)halides in toluene did not offer any hint of adduct formation by NMR in the temperature range of −80 to +70 °C, which contrasts with the readily accessible [(PCy_3_)_2_Pt → ZnBr_2_].^10^ Considering that the basicity of **1** may be superior to that of [Pt(PCy_3_)_2_], we ascribe the absence of MOLP formation from the former to steric reasons. Switching to dichloromethane, fluorobenzene, and tetrahydrofuran to improve the solubility of the zinc salt did not alter these results. However, addition of 1 equiv of the more acidic Zn(C_6_F_5_)_2_ to a colorless C_6_D_6_ solution of **1** caused instant coloration to bright yellow. Multinuclear NMR spectroscopic analysis suggested formation of the bimetallic adduct [(P^*t*^Bu_3_)_2_Pt → Zn(C_6_F_5_)_2_] (**2** in Scheme 1). The most distinctive feature is a pronounced decrease in the ^1^*J*_PPt_ coupling constant to a value of 3328 Hz (δ = 93.1 ppm; c.f. **1**: δ = 100.2 ppm, ^1^*J*_PPt_ = 4410 Hz), a common symptom of MOLP formation in Pt(0) compounds due to the reduced s character of the Pt–P bonds in the bimetallic adduct.^9,12^ Alongside this, a new set of ^19^F{^1^H} resonances at −115.7, −157.4, and −162.0 ppm (c.f. Zn(C_6_F_5_)_2_: δ = −118.0, −152.5, and −160.5 ppm) was recorded.

**Figure 1:**
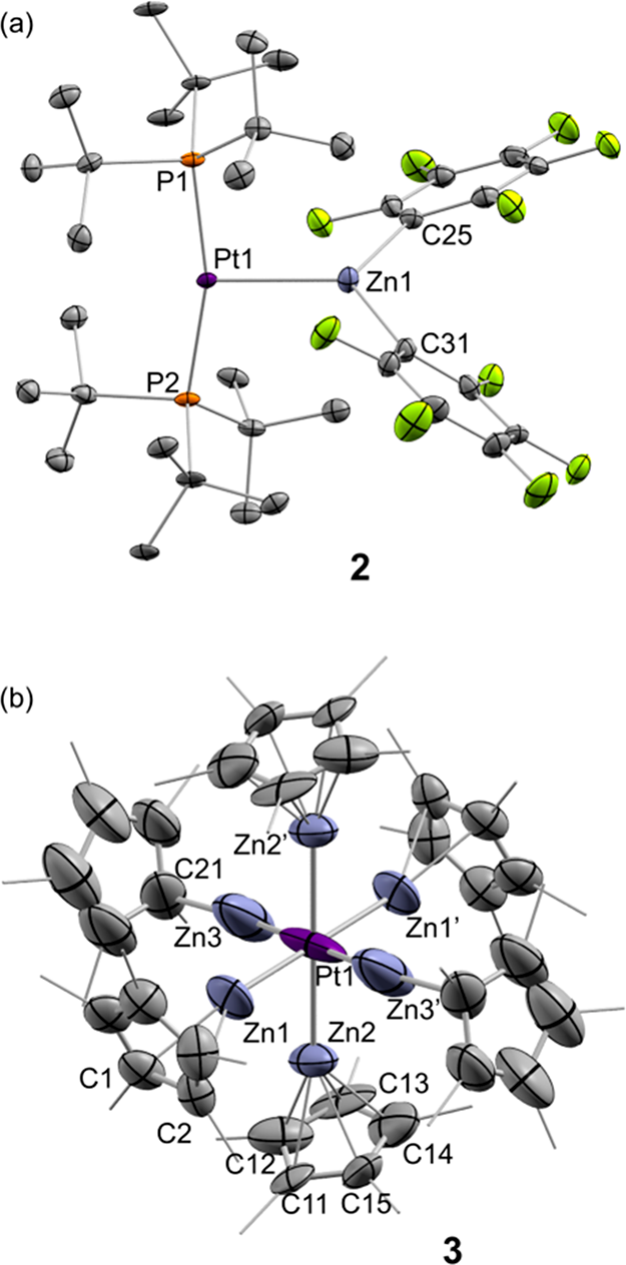
ORTEP diagram of compounds **2** and **3**. Hydrogen atoms have been excluded, and methyl groups of Cp* ligands are represented in wire-frame format for clarity. Thermal ellipsoids are set at 50% probability.

The molecular structure of **2** was authenticated by single-crystal X-ray diffraction studies (Figure 1a) confirming the proposed bimetallic formulation. This represents the first example of a Pt(0)/organozinc MOLP. It exhibits a T-shaped geometry around the platinum center, slightly distorted due to the steric pressure exerted by the *tert*-butyl groups in close proximity to the perfluorinated aryl rings (P–Pt–P = 165.32(4)°). As in other bisphosphine Pt(0)-based MOLPs, the Pt–P bond distances (2.325 Å on average) are modestly elongated with respect to that of precursor **1** (2.25 Å).^14^ The Pt–Zn bond length (2.4663(6) Å) is shorter than in the related [(phen)Ar_2_Pt^II^ → Zn(C_6_F_5_)_2_] (phen = phenanthroline) adduct, which contains a less basic Pt(II) donor (2.5526(5) Å),^6^ and just marginally longer than that in [(Cy_3_P)_2_Pt → ZnBr_2_] (2.4040(6) Å).^10^ Steric constraints in **2** force the perfluorphenyl rings to bend away from the platinum center, with the C25—Zn–C31 angle of 117.73(18)° being significantly reduced compared to those of Zn(C_6_F_5_)_2_ (172.6°)^15^ and even [(phen)Ar_2_Pt^II^ → Zn(C_6_F_5_)_2_] (134.8°).^6^

At variance with its fluorinated analogue, the less acidic ZnPh_2_ does not react with **1**, as monitored by variable temperature NMR and visually inferred by the colorless appearance of the reaction mixture even after prolonged periods of time. Similarly, no Pt → Zn interactions were detected upon addition of 10 equiv of ZnR_2_ (R = Me, Et, and η^5^-C_5_Me_5_) to C_6_D_6_ solutions of **1**, again pointing out the need for a highly electrophilic Zn center to overcome the distortion of the linear Pt(0) precursor to accommodate the bimetallic dative bond. Next, we examined the reactivity of **1** with the Zn(I) dimer [Zn_2_Cp*_2_]^13^ in light of its capacity to form zinc-rich polymetallic complexes with transition metal precursors.^16^ For instance, Fischer has investigated the reactivity between [Zn_2_Cp*_2_] and low-valent M(0) precursors (M = Ni, Pd, and Pt), recurrently identifying the homolytic cleavage of the Zn–Zn bond by insertion of the transition metal.^17^

**Scheme 2:**
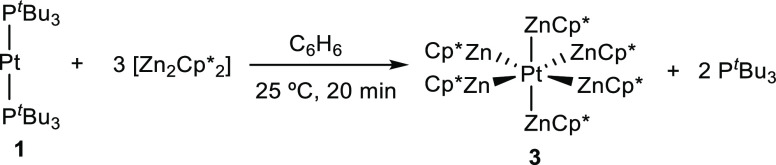
Synthesis of Compound **3** by the Reaction between **1** and [Zn_2_Cp*_2_]

^31^P{^1^H} NMR monitoring of an equimolar mixture of **1** and [Zn_2_Cp*_2_] in C_6_D_6_ showed the release of free phosphine (δ = 63.0 ppm) without any other detectable intermediate. It soon became evident that a 3-fold excess of the Zn(I) dimer was required to achieve complete consumption of **1**. Under these conditions, the highly unstable compound [Pt(ZnCp*)_6_] (**3**) forms as the major species (ca. 80% NMR yield) by insertion of the Pt center into the Zn–Zn bonds of three molecules of [Zn_2_Cp*_2_]. (Scheme 2). Compound **3** slowly precipitates as bright orange crystals, which allowed us to ascertain its heptametallic structure by X-ray diffraction analysis (Figure 1b). It can be described as an unusual 16-electron octahedral complex in which each vertex is occupied by a neutral 1-electron ZnCp* ligand. This is in stark contrast with all prior Zn-rich polymetallic compounds of late transition metals, which consistently fulfill the 18 valence electron rule.^16^ The steric shrouding provided by the six planar cyclopentadienyl ligands stabilizes the somewhat encapsulated electron-rich platinum center.

The solid-state structure shows three pairs of ZnCp* ligands that differ slightly from each other in terms of Zn coordination. Two of these fragments present η^5^-coordination (*d*_Zn–C_ ≈ 2.24–2.37 Å), and a second pair binds to the Cp* in an η^2^-fashion. The third pair exhibits an η^1^-binding (shortest *d*_Zn3–C21_ = 2.06(3) Å; the rest are >2.6 Å). While the former two pairs present Pt–Zn bond distances (2.419(3) and 2.401(4) Å) comparable to prior examples,^17^ the η^1^-bound ZnCp* fragment displays a Pt–Zn bond (2.238(7) Å) shortened by 0.34 Å with respect to the sum of the covalent radii (2.58 Å).^18^ In THF-*d*_8_ solution, a single ^1^H NMR resonance at 1.92 ppm indicates rapid dynamic exchange among the possible conformations of the ZnCp* ligands. In fact, low temperature NMR (up to −80 °C) was insufficient to freeze the dynamic process.

**Figure 2:**
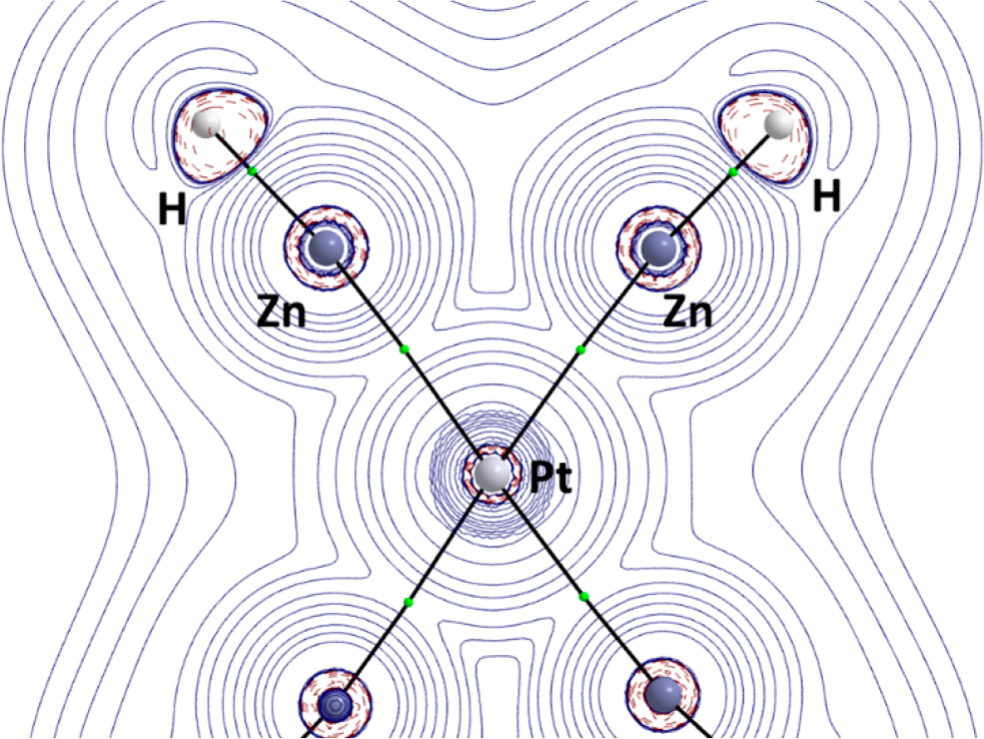
Contour line diagrams ∇^2^ρ(r) for Pt(ZnH)_6_ in the Zn–Pt–Zn plane. The solid lines connecting the atomic nuclei are the bond paths, while the small green spheres indicate the corresponding bond critical points.

Compound **3** strongly resembles the closed-shell 18-electron [Pt(ZnCp*)_4_(ZnR)_4_] species (R = Me and Et) described by Fischer and co-workers.^17b^ The latter compounds exhibit rather long Zn···Zn distances (ranging from 2.812 to 3.115 Å), which have been regarded as noninteracting or only weakly interacting.^17e^ Similar Zn···Zn distances were found in **3** (>3.0 Å). To confirm the negligible interaction between the zinc centers in **3**, we computationally explored the topology of the model system Pt(ZnH)_6_, analogous to the model Pt(ZnH)_8_ used by Fischer and Frenking to understand the bonding situation in [Pt(ZnCp*)_4_(ZnR)_4_],^17b,17e^ using the QTAIM (Quantum Theory of Atoms in Molecules) method (see computational details in the Supporting Information). Figure 2 shows the Laplacian distribution of Pt(ZnH)_6_ computed in the Zn–Pt–Zn plane. As expected, bond critical points (BCPs) together with their associated bond paths (BPs) are found between the zinc and platinum centers (computed Pt–Zn bond distances ∼ 2.47 Å). In contrast, no BCPs or BPs were located between the zinc atoms (computed Zn···Zn bond distances ranging from 2.91 to 2.93 Å), which similar to Pt(ZnR)_8_^17b,17e^ supports the above-commented noninteracting nature of Zn···Zn in **3**.

More quantitative insight into the bonding situation in **3** can be obtained by means of the energy decomposition analysis (EDA) method, also used by Fischer, Frenking, and co-workers to analyze the bonding in the analogous Pt(ZnH)_8_ (*D*_4*d*_).^17b^ Thus, we compare the EDA data for Pt(ZnH)_6_ and Pt(ZnH)_8_ using the same partitioning scheme reported previously, namely, Pt(0) and (ZnH)_*n*_ (*n* = 6 and 8) in their singlet states as fragments. Table 1 gathers the corresponding EDA values computed at the ZORA-BP86-D3/TZ2P//BP86-D3/def2-TZVPP level including the original data reported previously for Pt(ZnH)_8_ (*D*_4*d*_) computed at the rather similar ZORA-BP86/TZ2P//RI-BP86/def2-TZVPP level. From the data in Table 1, the resemblance between both Pt(0) compounds becomes evident. Although the computed interaction energy, Δ*E*_int_, is higher in Pt(ZnH)_8_ (which is not surprising as the Pt center interacts with two additional one-electron ZnH ligands), in both cases, the platinum atom bears a small negative charge, which is consistent with the chosen neutral fragments. Despite that, the main contribution to the bonding comes from the electrostatic interactions, representing ca. 76–77% of the total attractions. The contribution resulting from orbital interactions (mainly involving the d atomic orbitals of platinum) is significantly much lower and those coming from dispersion interactions can be considered as negligible. This therefore indicates that the bonding in newly prepared compound **3** (and the analogous [Pt(ZnCp*)_4_(ZnR)_4_]) can be viewed mainly as a result of the electrostatic interactions between the platinum center and the surrounding ZnCp* ligands.

**Table 1:** EDA results at ZORA-BP86-D3/TZ2P for [Pt(ZnH)*_n_*] (*n* = 6 and 8) with the fragments M(s^0^d^10^) and (ZnH)_*n*_ in the Singlet State

compound	Pt(ZnH)_6_	Pt(ZnH)_8_	Pt(ZnH)_8_^a^
Δ*E*_int_	–234.1	–288.9	–279.0
Δ*E*_Pauli_	434.7	468.8	486.0
Δ*E*_elstat_^b^	–518.4 (77.5%)	–575.6 (76.0%)	–583.4 (76.3%)
Δ*E*_orb_^b^	–145.5 (21.8%)	–175.2 (23.1%)	–181.6 (23.7%)
Δ*E*_disp_^b^	–4.8 (0.7%)	–6.9 (0.9%)	
*q*(Pt)^c^	–0.21	–0.21	

a Energy values (kcal/mol) taken from ref (17b).

b Percentage values in parentheses give the contributions to the total attractive energy Δ*E*_elstat_ + Δ*E*_orb_ + Δ*E*_disp_.

c Computed Hirshfeld charges at the platinum center.

**Scheme 3:**
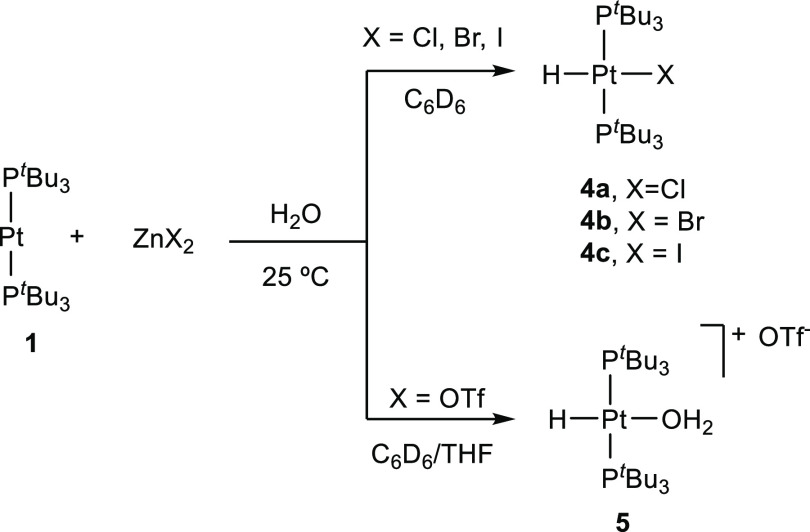
Activation of Polar O–H Bond by Pt(0)/Zn(II) FLPs

We next interrogated the ability of these Pt/Zn bimetallic pairs to activate both polar and nonpolar bonds using water and dihydrogen as model substrates. We mainly directed our efforts toward pairs containing inorganic zinc salts, as organozinc compounds (**1**/ZnR_2_, **2** and **3**) were rapidly hydrolyzed in the presence of water. Besides, those species remained inactive toward H_2_ under all attempted conditions. In contrast, equimolar benzene suspensions of **1** and ZnX_2_ (X = Cl, Br, I, and OTf) readily react with H_2_O (5 equiv) by means of O–H bond activation (Scheme 3).^11,12^ It is important to remark that **1** does not react with water on its own even under more forcing conditions (80 °C, 24 h). However, in the presence of zinc halides formation of *trans*-[PtHX(P^*t*^Bu_3_)_2_] (X = Cl, Br, and I; **4**, Scheme 3) is evidenced by a distinctive low-frequency ^1^H NMR resonance due to the metal hydride (δ = −19.2 (**4a**, Cl), −18.4 (**4b**, Br), and −16.4 (**4c**, I) ppm), exhibiting scalar coupling to both ^31^P (^2^*J*_HP_ ≈ 12 Hz) and ^195^Pt (^1^*J*_HPt_ ≈ 1100 Hz) nuclei. In the case of Zn(OTf)_2_, the reduced coordinating capacity of the triflate moiety compared to halide anions led to the cationic hydride-aquo complex *trans*-[PtH(OH_2_)(P^*t*^Bu_3_)_2_]^+^(**5**) as the only observable product. Formation of compounds **4** and **5** is accompanied by the appearance of a fine precipitate of zinc hydroxide salts.

**Scheme 4:**
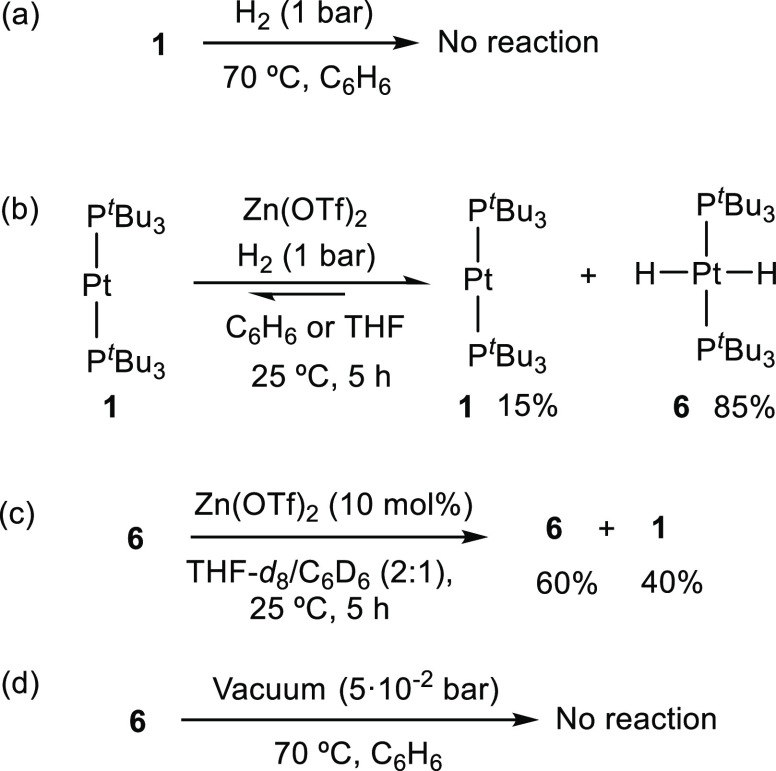
Reactivity of Bimetallic Pair **1**/Zn(OTf)_2_ with H_2_

As mentioned briefly above, water activation by combining **1** with transition metal Lewis acids [Cu(CH_3_CN)_4_]PF_6_^11^ and AgNTf_2_,^12^ has recently been reported. Formation of an intermediate characterized by a Pt → M dative interaction is proposed as the initial step in both cases, after which the cooperative cleavage of the O–H bond takes place. Our experiments indicate that bimetallic adduct formation is not favored for zinc salts; thus, an FLP-type mechanism seems more likely. In fact, we have already demonstrated that compound **1** acts as a Lewis basic site in bimetallic FLPs by partnering it with sterically crowded Au(I) compounds.^19^ Our prior mechanistic investigations allowed us to conclude that those Pt(0)/Au(I) pairs mediate the cleavage of the H–H bond in dihydrogen by a genuine FLP mechanism.^19b^ We wondered if the same would apply for the Pt/Zn pairs investigated herein. Once again, it is worth mentioning that neither **1** nor zinc (pseudo)halides react with H_2_ on their own (Scheme 4a). Similarly, the combination of **1** and zinc halides in benzene or THF did not provide any reactivity upon exposure to H_2_ (2 bar, 70 °C). However, in the presence of the more acidic Zn(OTf)_2_, dihydrogen activation proceeds smoothly to generate Pt(II) dihydride **6**(20) even under mild conditions (H_2_ 1 bar, 25 °C, 5 h; Scheme 4b). Compound **6** is produced in ca. 85% spectroscopic yield, exhibiting a characteristic ^1^H NMR resonance at −2.91 ppm (^2^*J*_HP_ = 16.4 Hz, ^1^*J*_HPt_ = 780.6 Hz).

Formation of **6** suggests a catalytic role of Zn(OTf)_2_ during the hydrogenation of **1**, which has previously been observed for the hydrogenation of imines catalyzed by the same zinc species.^21^ In fact, decreasing the amount of Zn(OTf)_2_ to only 5 mol % with respect to **1** under otherwise identical conditions led to the formation of **6** in comparable yields. In fact, the amount of zinc and the nature of the solvent did not have any apparent influence on the extent of dihydride produced, which was obtained in yields between 80 and 90% in all cases. Attempts to reach full hydrogenation of **1** were unsuccessful despite longer reaction times, higher temperatures and increasing loadings of zinc. These observations imply that hydrogenation of **1** is a reversible process. We confirmed this idea by adding Zn(OTf)_2_ (10 mol %) to a THF-*d*_8_/C_6_D_6_ (2:1) solution of dihydride **6** in a sealed NMR tube (Scheme 4c). Reaction monitoring evidenced evolution to a mixture of both **1** and **6** in a ca. 2:3 ratio after 5 h, as well a minute amount of free H_2_ identified by an ^1^H NMR peak at 4.42 ppm. Replacing the atmosphere by H_2_ (1 bar) led to **6** in around 85% yield. The presence of zinc is also essential for dehydrogenation, since in its absence the release of H_2_ could not be detected even by heating **6** under dynamic vacuum (70 °C, 50·mbar, Scheme 4d). This process resembles both the dehydrogenation of [PtH_2_(PCy_3_)_2_] promoted by C_60_,^22^ as well as the role played by Zn(C_6_F_5_)_2_ in facilitating biaryl reductive elimination from Zn(II) complexes.^6^

**Scheme 5:**
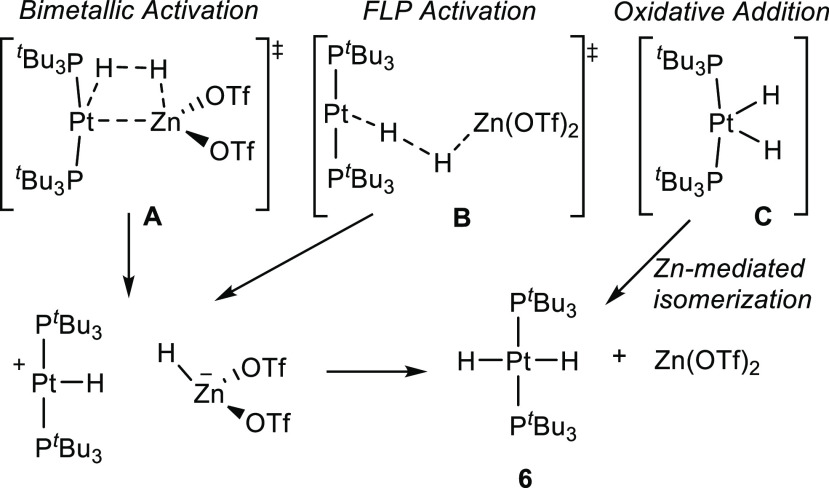
Potential Mechanisms for H_2_ Activation by **1**/Zn(OTf)_2_

The mechanism of reversible heterolytic dihydrogen splitting holds great interest due to its connection to hydrogen production and the action of hydrogenase enzymes. It has also been largely studied as a benchmark transformation to gauge FLP behavior and, despite its apparent simplicity, remains a topic of intense research.^23^ In this line, the absence of adduct formation from the pair **1**/Zn(OTf)_2_ along with its cooperative bond activation could be understood in terms of FLP principles.^24^ We performed several experiments to gain some preliminary mechanistic information. First, we determined the kinetic isotopic effect (KIE) for H_2_ versus D_2_ splitting, which has a strong inverse value of 0.59 ± 0.1 (see the Supporting Information for details). This is an uncommon finding^25^ that compares well with our previously reported Pt(0)/Au(I) bimetallic FLP (KIE = 0.46 ± 0.04), where a genuine frustrated mechanism was ascertained.^19b^ We postulated that the origin for such a strong inverse KIE derived from an FLP productlike transition state whose bimetallic structure offered an assortment of H-containing bending modes that contribute to the zero-point energy (ZPE). We anticipate that a similar transition state in the present system (**B** in Scheme 5) would analogously derive in a strong inverse KIE, as observed experimentally. Direct oxidative addition of dihydrogen over **1** to form *cis*-[PtH_2_(P^*t*^Bu_3_)_2_] followed by Zn-assisted isomerization^4^ could be considered an alternative mechanism (**C** in Scheme 5). However, solutions of **1**/Zn(OTf)_2_ catalyze rapid (*t*_1/2_ < 15 min) exchange between H_2_ and D_2_ to produce HD (δ = 4.36 ppm, ^1^*J*_HD_ = 42.6 Hz) in a statistical amount, which seems to disfavor a classical oxidative addition route. In fact, the individual monometallic species mediate the exchange at a considerable slower pace (*t*_1/2_ > 2 days). Interestingly, compound [PtH(P^*t*^Bu_3_)_2_]^+^, which would be an intermediate during FLP-type H_2_ activation, promotes H/D scrambling at a rate comparable to the bimetallic pair. This agrees with its existence as a transient intermediate during the hydrogenation of **1**, thus supporting the idea of a bimetallic FLP mechanism (through **B** in Scheme 5). Nevertheless, these preliminary experiments cannot yet rule out a more traditional bimetallic H_2_ activation route implying a transient dative Pt → Zn bond (**A** in Scheme 5) or the active participation of triflate substituents.^26^

ConclusionsIn summary, we report the formation of two new Pt/Zn polymetallic complexes. While the metal-only Lewis adduct [(P^*t*^Bu_3_)_2_Pt → Zn(C_6_F_5_)_2_] (**2**) represents the first Pt(0)/organozinc MOLP, the reaction between [Pt(P^*t*^Bu_3_)_2_] (**1**) and [Zn_2_Cp*_2_] yields the hexametallic, homoleptic compound [Pt(ZnCp*)_6_] (**3**). At variance with previous Zn-rich polymetallic compounds, the latter does not fulfill the 18 valence electron rule, since it is considered an octahedral 16-electron species. While these complexes remain inactive toward dihydrogen, pairing **1** with Zn(OTf)_2_ results in cooperative dihydrogen cleavage. Preliminary kinetic and isotopic exchange experiments support a bimetallic FLP-type mechanism. Similarly, the activation of O–H bonds in water proceeds readily in the presence of Pt/Zn pairs, while the individual components reveal no activity.

## Experimental Section

### General Considerations

All preparations and manipulations were carried out using standard Schlenk and glovebox techniques, under an atmosphere of argon and of high purity nitrogen, respectively. All solvents were dried, stored over 3 Å molecular sieves, and degassed prior to use. Toluene (C_7_H_8_) and *n*-pentane (C_5_H_12_) were distilled under nitrogen over sodium. Tetrahydrofuran (THF) and diethyl ether were distilled under nitrogen over sodium/benzophenone. (D_6_)Benzene was dried over molecular sieves (3 Å), and (D_8_)THF was distilled under argon over sodium/benzophenone, and CD_2_Cl_2_ and fluorobenzene over CaH_2_ distilled under argon. Compounds **1**,^27^ ZnPh_2_,^28^ [Zn_2_Cp*_2_],^29^ and [PtHCl(PtBu_3_)_2_]^30^ were prepared as described previously. Other chemicals were commercially available and used as received. Solution NMR spectra were recorded on Bruker AMX-300, DRX-400 and DRX-500 spectrometers. Spectra were referenced to external SiMe_4_ (δ: 0 ppm) using the residual proton solvent peaks as internal standards (^1^H NMR experiments) or the characteristic resonances of the solvent nuclei (^13^C NMR experiments), while ^31^P was referenced to H_3_PO_4_ and ^19^F to CFCl_3_. Spectral assignments were made by routine one- and two-dimensional NMR experiments where appropriate. For elemental analyses, a LECO TruSpec CHN elementary analyzer was utilized. The supplementary crystallographic data for this paper has been deposited in the Cambridge Crystallographic Data Centre with codes 2062801 and 2062802.

### Compound **2**

To a mixture of [Pt(P^*t*^Bu_3_)_2_] (**1**) (50 mg, 0.083 mmol) and Zn(C_6_F_5_)_2_ (33 mg, 0.083 mmol) was added 5 mL of toluene, and the solution was stirred for 30 min, then kept at −30 °C. Orange crystals of **2** were collected and washed with cold pentane (43 mg, 52%). Anal. Calcd for C_36_H_54_F_10_P_2_PtZn: C, 43.3; H, 5.5. Found: C, 43.0; H, 5.7. ^1^H NMR (400 MHz, C_6_D_6_, 25 °C) δ: 1.28 (vt, 54 H, ^3^*J*_HP_ = 6.3 Hz, ^t^Bu). ^13^C{^1^H} NMR (100 MHz, CD_2_Cl_2_, 25 °C) δ: 148.6 (br d, ^1^*J*_CF_ = 227 Hz, *o*-C_6_F_5_), 139.8 (br d, ^1^*J*_CF_ = 232 Hz, *p*-C_6_F_5_), 136.6 (br d, ^1^*J*_CF_ = 254 Hz, *m*-C_6_F_5_), 128.2 (br, ipso-C_6_F_5_), 40.2 (vt, ^1^*J*_CP_ = 8 Hz, Pt–P(*C*(CH_3_)_3_), 33.0 (Pt–P(C(*C*H_3_)_3_). ^31^P{^1^H} NMR (160 MHz, C_6_D_6_, 25 °C) δ: 93.1 (^1^*J*_PPt_ = 3328 Hz). ^19^F{^1^H} NMR (376 MHz, C_6_D_6_, 25 °C) δ: −115.7, −157.4, −162.0.

### Compound **3**

To a mixture of complex [Pt(P^*t*^Bu_3_)_2_] (**1**) (50 mg, 0.083 mmol) and [Zn_2_Cp*_2_] (99 mg, 0.249 mmol) was added 3 mL of benzene. The solution was stirred for 20 min at room temperature. Complex **3** crystallized from the crude reaction after 12 h (34 mg, 30%). Anal. Calcd for C_60_H_90_PtZn_6_: C, 51.5; H, 6.5. Found: C, 51.5; H, 6.8. ^1^H NMR (400 MHz, C_6_D_6_, 25 °C) δ: 1.45 (Me). ^13^C{^1^H} NMR (100 MHz, THF-*d*_8_, 25 °C) δ: 112.0 (*C*_5_Me_5_), 12.0 (C_5_*Me*_5_).

## Supporting Information Available

Synthetic procedures, kinetic studies, X-ray structural data, computational details, and NMR spectra (PDF)
Cartesian coordinates (XYZ)

## Supplementary Material

om1c00088_si_001.pdf

om1c00088_si_002.xyz

## Accession Codes

CCDC 2062801 and 2062802 contain the supplementary crystallographic data for this paper. These data can be obtained free of charge via www.ccdc.cam.ac.uk/data_request/cif, or by emailing data_request@ccdc.cam.ac.uk, or by contacting The Cambridge Crystallographic Data Centre, 12 Union Road, Cambridge CB2 1EZ, UK; fax: +44 1223 336033.
