# Supplementary material for: Reactivity of [Pt(PtBu3)2] with Zinc(I/II) Compounds: Bimetallic Adducts, Zn–Zn Bond Cleavage, and Cooperative Reactivity
Source: Organometallics. 2021 Apr 13;40(8):1113–9. doi: 10.1021/acs.organomet.1c00088 (PMC8479860; doi:10.1021/acs.organomet.1c00088)
Supplement: Supplementary file 1 — om1c00088_si_001.pdf [file om1c00088_si_001.pdf]

Reactivity of [Pt(P^tBu_3)$_2$] with Zinc(I/II) Compounds: Bimetallic Adducts, Zn—Zn Bond Cleavage and Cooperative Reactivity

Nereida Hidalgo[a], Carlos Romero-Pérez[a], Celia Maya[a], Israel Fernández[b], Jesús Campos[a]*

[a]*Instituto de Investigaciones Químicas (IIQ), Departamento de Química Inorgánica and Centro de Innovación en Química Avanzada (ORFEO-CINQA). Universidad de Sevilla and Consejo Superior de Investigaciones Científicas (CSIC). Avenida Américo Vespucio 49, 41092 Sevilla (Spain).*

[b]*Departamento de Química Orgánica I and Centro de Innovación en Química Avanzada (ORFEO-CINQA), Facultad de Ciencias Químicas, Universidad Complutense de Madrid, Madrid (Spain).*

**jesus.campos@iiq.csic.es*

SUPPORTING INFORMATION

1. Synthesis and characterization of compounds S2

2. Kinetic studies S4

3. Isotopic exchange experiments S4

4. X-Ray structural characterization of new compounds S5

5. Computational details S7

6. NMR spectra of new compounds. S8

7. References S12

1. **Synthesis and characterization of compounds.**

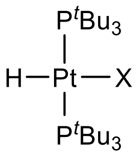

4a, X=Cl; **4b**, X = Br; **4c**, X = I

Compounds 4. The synthesis of compounds **4** were carried out using the same experimental procedure. A solution of compound **1** (20 mg, 0.033 mmol) in C_6D_6 (0.5 mL) was added over 1 eq. of the corresponding zinc halide, ZnX_2 (X = Cl, Br, I) in a *J. Young* NMR tube. The suspension was shaken and then water (3 µL, 0.165 mmol) was added and the progress of the reaction monitored by ^{1}H and ^{31}P{^{1}H} NMR spectroscopy. Compounds **4a-c** were formed in >90% spectroscopic yield after one hour at 25 ºC. ^{1}H and ^{31}P{^{1}H} NMR spectroscopic details match perfectly with those previously reported.[1]

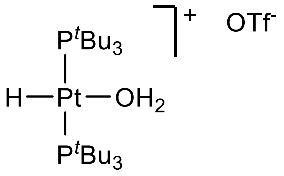

Compound 5. A solution of [Pt(P^{t}Bu$_3$)$_2$] (**1**) (20 mg, 0.033 mmol) in C_6D_6/THF (0.2/0.4 mL) was added over $Zn(OTf)_2$ (12 mg, 0.033 mmol) in a *J. Young* NMR tube. The solution was shaken and then water (3 µL, 0.165 mmol) was added. Compound **5** was formed in around 85% spectroscopic yield after 10 minutes at 25 ºC. ^{1}H and ^{31}P{^{1}H} NMR spectroscopic details match perfectly with those previously reported.[2]

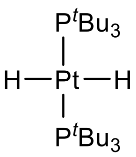

Compound 6. A solution of compound **1** (20 mg, 0.033 mmol) in THF/C_6D_6 (0.4/0.2 mL) was added over $Zn(OTf)_2$ (12 mg, 0.033 mmol) in a *J. Young* NMR tube. The solution was shaken and the nitrogen atmosphere replaced by hydrogen (1 atm). Compound **6** was formed in around 85% spectroscopic yield after 5 hours at 25 ºC. ^{1}H and ^{31}P{^{1}H} NMR spectroscopic details match perfectly with those previously reported.[1]

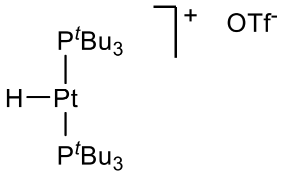

$[PtH(PtBu_3)_2]^+$. To a mixture of $[PtHCl(PtBu_3)_2]$ (50 mg, 0.079 mmol) and AgOTf (20 mg, 0.079 mmol) was added toluene (8 mL) under N_2 atmosphere. The reaction was covered with aluminum foil and stirred at room temperature for 1 hour. The solution was filtered and the volatiles were removed in vacuo to yield the compound as a white powder (42 mg, 71%). ^{1}H and ^{31}P{^{1}H} NMR spectroscopic details match perfectly with those previously reported.[2]

2. Kinetic studies.

Kinetic studies were carried out to determine the kinetic isotopic effect (KIE) of dihydrogen activation by compound **1** and $Zn(OTf)_2$. In a *J. Young* NMR tube, a mixture of compounds **1** (5 mg, 0.008 mmol) and $Zn(OTf)_2$ (3 mg, 0.008 mmol) was dissolved in THF-d_8/C_6D_6 (0.4/0.2 mL) at room temperature. The nitrogen atmosphere was replaced by either H_2 or D_2 (1 bar) and the solution was shaken. The progress of the reaction was monitored by $^{31}P\{^1H\}$ NMR spectroscopy at 25 ºC by means of the disappearance of compound **1** and using triphenylphosphine oxide as internal standard.

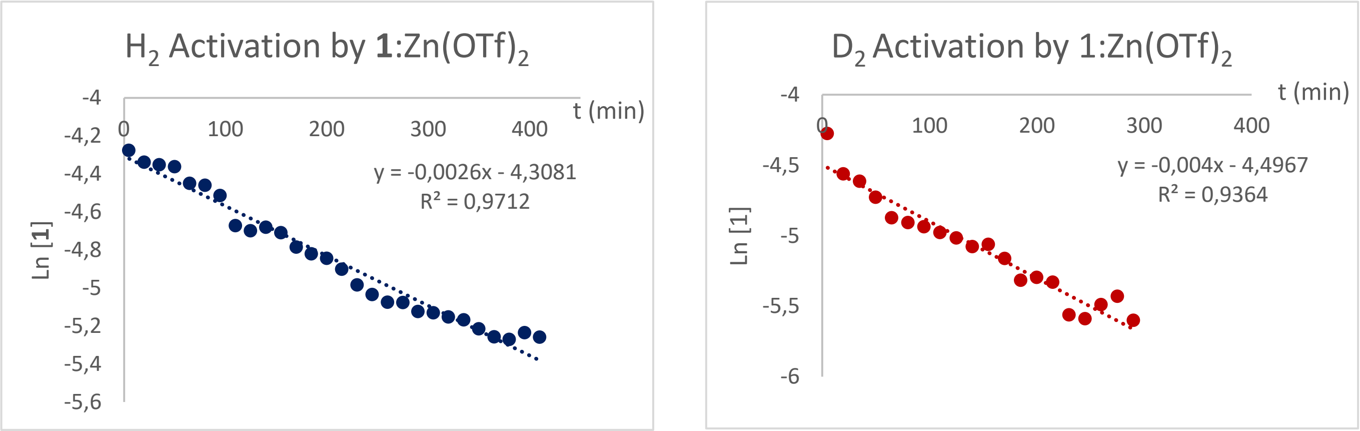

Figure S1. Representative examples of the kinetic profiles for the activation of H_2 or D_2 by the **1**:$Zn(OTf)_2$ system.

3. Isotopic exchange experiments

A solution of $[Pt(P^tBu_3)_2]$ (**1**) (5 mg, 0.008 mmol) and $Zn(OTf)_2$ (3 mg, 0.008 mmol) was dissolved in THF-d_8/C_6D_6 (0.4/0.2 mL) and a H_2/D_2 mixture (1:1, 1 bar) was added in a *J. Young* NMR tube. The reaction was monitored by ^1H NMR NMR spectroscopy at 25 ºC to track the progress of HD evolution. To complete these studies, the same procedure was performed with $[Pt(^tBu_3)_2]$, $Zn(OTf)_2$ and $[PtH(PtBu_3)_2]^+$, separately.

4. X-Ray structural characterization of new compounds.

Crystallographic details. Single crystals of suitable size of each compound were selected and covered with FOMBLIN oil and mounted on a glass fiber. Data collections have been performed on a Bruker SMART APEX II diffractometer with a CCD area detector on a D8 goniometer at 100 K, using graphite-monochromated and 0.5 mm-Monocap-collimated Mo-K_{α} radiation (λ=0.71073 Å).

Data collections were processed with APEX-W2D-NT (Bruker, 2004), cell refinement and data reduction with SAINT-Plus (Bruker, 2004) and the absorption was corrected by multiscan method applied by SADABS.[3] The space-group assignment was based upon systematic absences, E statistics, and successful refinement of the structure. The structure was solved by direct methods and expanded through successive difference Fourier maps, F^2 (SHELXTL).[4] Nonhydrogen atoms were refined anisotropically and hydrogen atoms connected to carbon atoms were included in idealized positions using a riding model for their refinement.

For structure **3**, crystals diffracted quite weakly at high angle. Many attempts were made to obtain better quality data, however crystal quality has always been very poor due to the very high instability of the complex towards air and moisture, as well as the low crystallographic stability of the structure itself due to solvent loss. A full set of data could be collected, however data above 0.90 angstroms was dominated by noise [I/sigma(I) < 1.0] and they have been omitted. For this reason, R1 and wR2 have high values. DFIX, SADI, DELU and SIMU commands have been used to retrain all the cyclopentyl groups. Zn3 atoms are disordered over two sites (Zn3A and Zn3B) the occupancies of which were constrained to sum to 1.0. Refinement showed residual electron density around Cp* bonded to Zn3 but this disorder could not be modeled. A summary of all crystallographic data and refinement parameters for each compound is provided in Table S1. Atomic coordinates, anisotropic displacement parameters and bond lengths and angles can be found in the cif files which have been deposited in the Cambridge Crystallographic Data Centre with no. 2062801-2062802. These data can be obtained free of charge from The Cambridge Crystallographic Data Centre via www.ccdc.cam.ac.uk/data_request/cif.

Table S1. Crystal data and structure refinement for compounds **2** and **3**.

	**2**	**3**
formula	$C_{39}H_{57}F_{10}P_2PtZn$	$C_{33}H_{48}Pt_{0.5}Zn_3$
fw	1038.24	738.37
cryst.size, mm	0.29 x 0.21 x 0.15	0.20 x 0.14 x 0.12
crystal system	Monoclinic	Triclinic
space group	$P2_1/c$	*P-1*
a, Å	12.5870(5)	12.3860(13)
b, Å	21.7560(10)	12.4280(12)
c, Å	16.8409(6)	12.9684(15)
α, deg	90	63.261(4)
β, deg	115.012(3)	66.832(5)
γ, deg	90	69.595(3)
V, Å^3	4179.3(3)	1602.8(3)
T, K	193(2)	193(2)
Z	4	2
ρ_{calc}, g cm^{-3}	1.650	1.530
μ, mm^{-1} (MoKα)	4.07	4.42
F(000)	2076	750
absorption corrections	multi-scan, 0.66-0.75	multi-scan, 0.59-0.75
θ range, deg	1.87 – 30.60	2.88 – 23.26
no. of rflns measd	81202	18599
R_{int}	0.097	0.053
no. of rflns unique	12753	4590
no. of params / restraints	496 / 0	351 / 262
R_1 ($I > 2\sigma(I)$) [a]	0.044	0.162
R_1 (all data)	0.077	0.191
wR_2 ($I > 2\sigma(I)$)	0.085	0.409
wR_2 (all data)	0.097	0.426
Diff.Fourier.peaks min/max, eÅ^{-3}	-1.992 / 3.120	-2.446/ 5.598
CCDC number	2062802	2062801

5. Computational Details.

Geometry optimization of compounds $Pt(ZnH)_6$ and $Pt(ZnH)_8$ (D_{4d}) were performed using the Gaussian16[5] suite of programs at the BP86[6]/def2-TZVPP[7] level of theory using the D3 dispersion correction suggested by Grimme et al.[8] This level is denoted BP86-D3/def2-TZVPP. All AIM results described in this work correspond to calculations performed at the BP86-D3/def2-TZVPP/WTBS(for Pt) level on the optimized geometry obtained at the BP86-D3/def2-TZVPP level. The WTBS (well-tempered basis sets)[9] have been recommended for AIM calculations involving transition metals.[10] The topology of the electron density was conducted using the AIMAll program package.[11]

The interaction between Pt(0) and $(ZnH)_n$ (n=6,8) has been investigated with the Energy Decomposition Analysis (EDA)[12] method. Within this approach, the interaction energy can be decomposed into the following physically meaningful terms:

$$\Delta E_{\text{int}} = \Delta E_{\text{elstat}} + \Delta E_{\text{Pauli}} + \Delta E_{\text{orb}} + \Delta E_{\text{disp}}$$

The term ΔE_{elstat} corresponds to the classical electrostatic interaction between the unperturbed charge distributions of the deformed reactants and is usually attractive. The Pauli repulsion ΔE_{Pauli} comprises the destabilizing interactions between occupied orbitals and is responsible for any steric repulsion. The orbital interaction ΔE_{orb} accounts for charge transfer (interaction between occupied orbitals on one moiety with unoccupied orbitals on the other, including HOMO–LUMO interactions) and polarization (empty-occupied orbital mixing on one fragment due to the presence of another fragment). Finally, the ΔE_{disp} term takes into account the interactions which are due to dispersion forces.

The EDA-NOCV calculations were carried out using the BP86-D3/def2-TZVPP optimized geometries with the program package ADF 2019.01[13] using the same functional (BP86-D3) in conjunction with a triple-ζ-quality basis set using uncontracted Slater-type orbitals (STOs) augmented by two sets of polarization function with a frozen-core approximation for the core electrons.[14] An auxiliary set of s, p, d, f, and g STOs were used to fit the molecular densities and to represent the Coulomb and exchange potentials accurately in each SCF cycle.[15] Scalar relativistic effects were incorporated by applying the zeroth-order regular approximation (ZORA).[16] This level of theory is denoted ZORA-BP86-D3/TZ2P//BP86-D3/def2-TZVPP.

6. NMR spectra of new compounds.

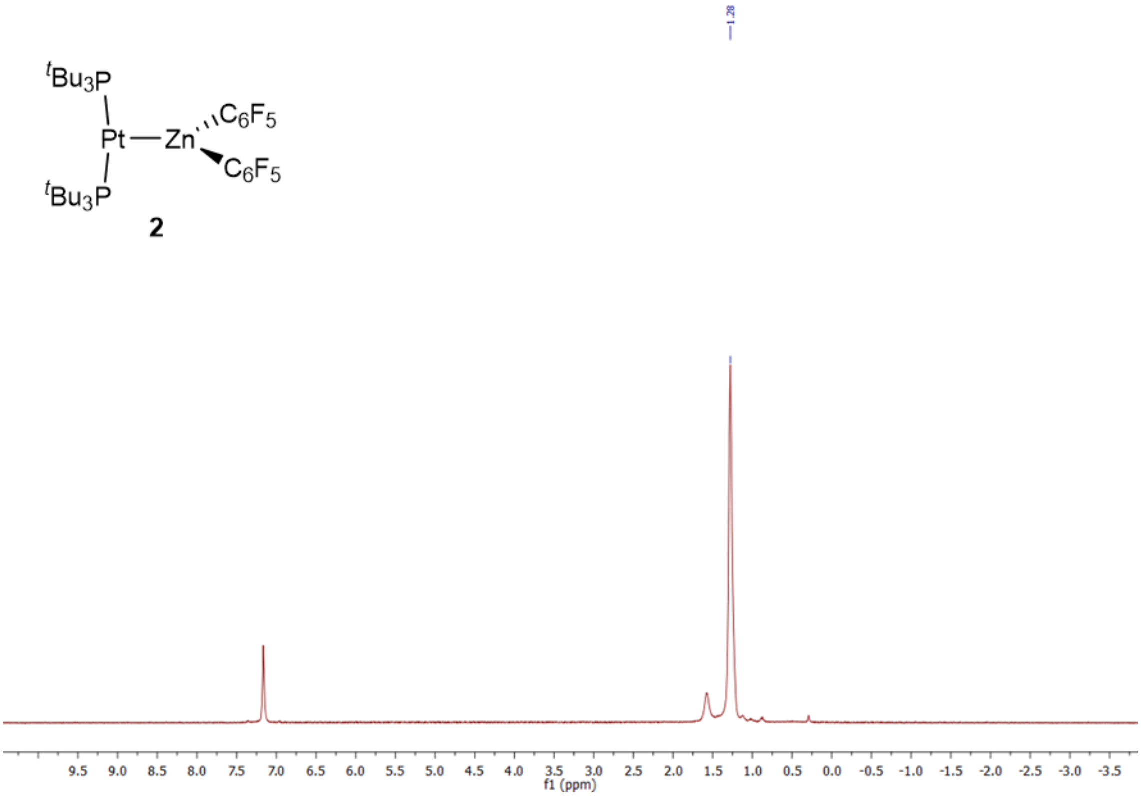

Figure S2. **^{1}H NMR** (400 MHz, C_6D_6, 25 ºC). Compound **2**.

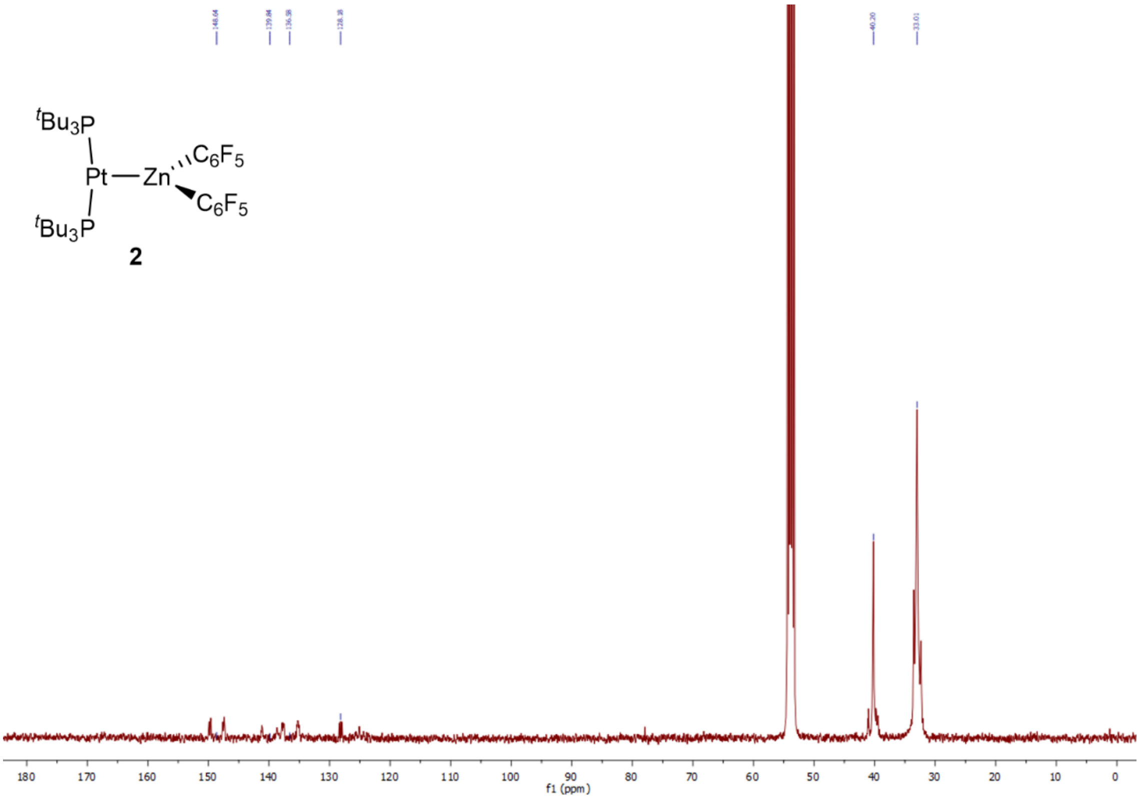

Figure S3. **^{13}C{^{1}H} NMR** (160 MHz, CD_2Cl_2, 25 ºC). Compound **2**.

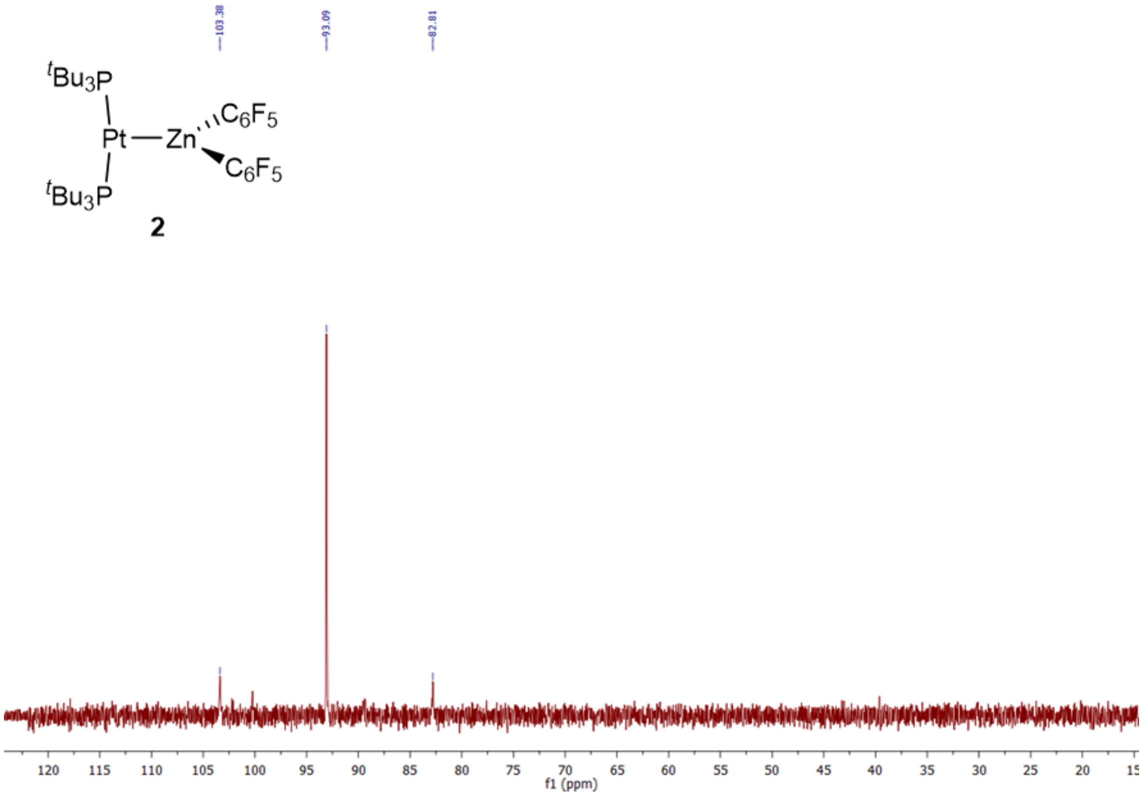

Figure S4. ^{31}**P{**^{1}**H} NMR** (160 MHz, C_6D_6, 25 ºC). Compound **2**.

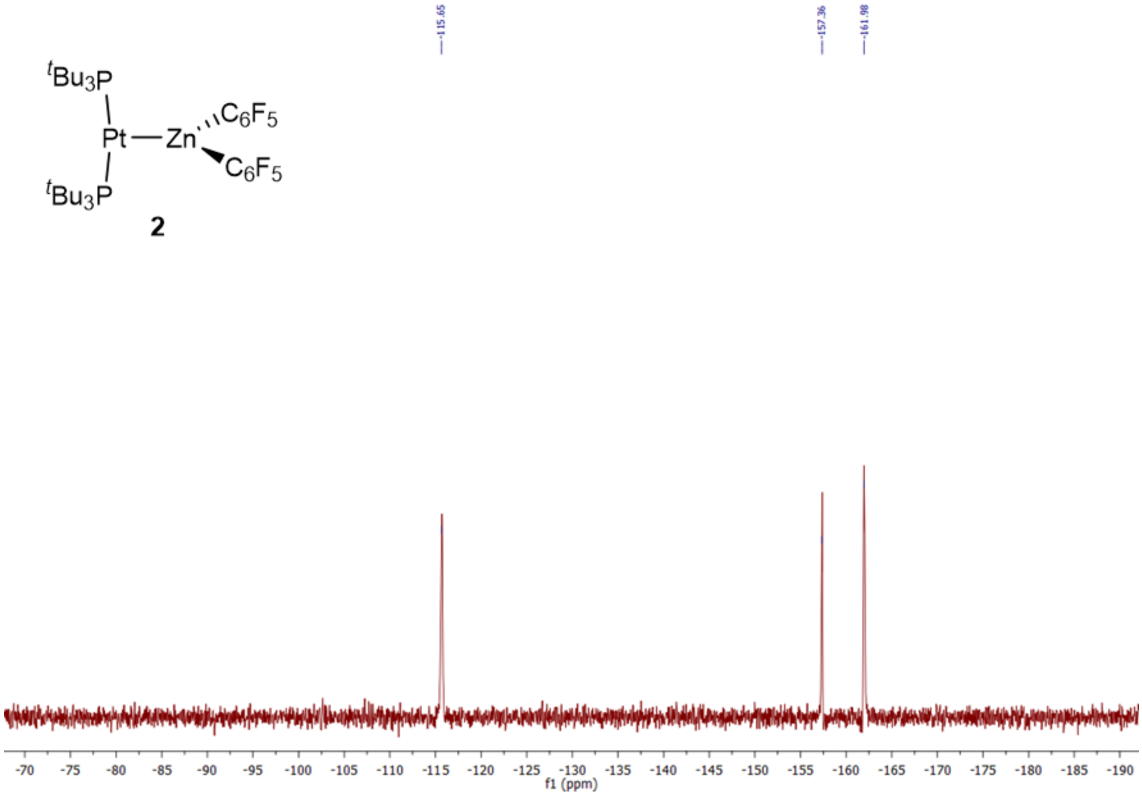

Figure S5. ^{19}**F{**^{1}**H} NMR** (376 MHz, C_6D_6, 25 ºC). Compound **2**.

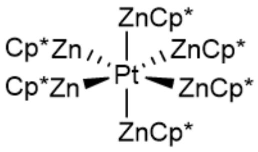

3

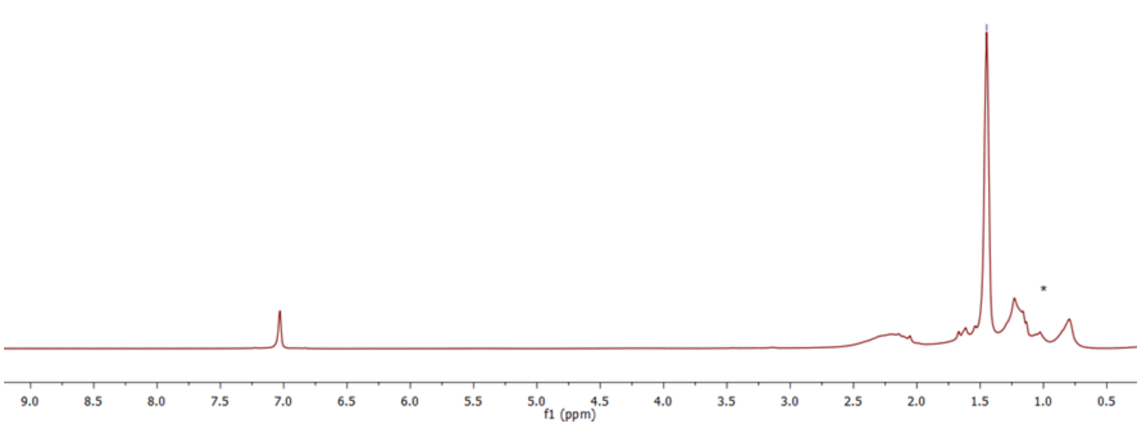

Figure S6. **^{1}H NMR** (400 MHz, C_6D_6, 25 ºC). Compound **3**. *Pentane.

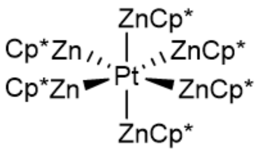

3

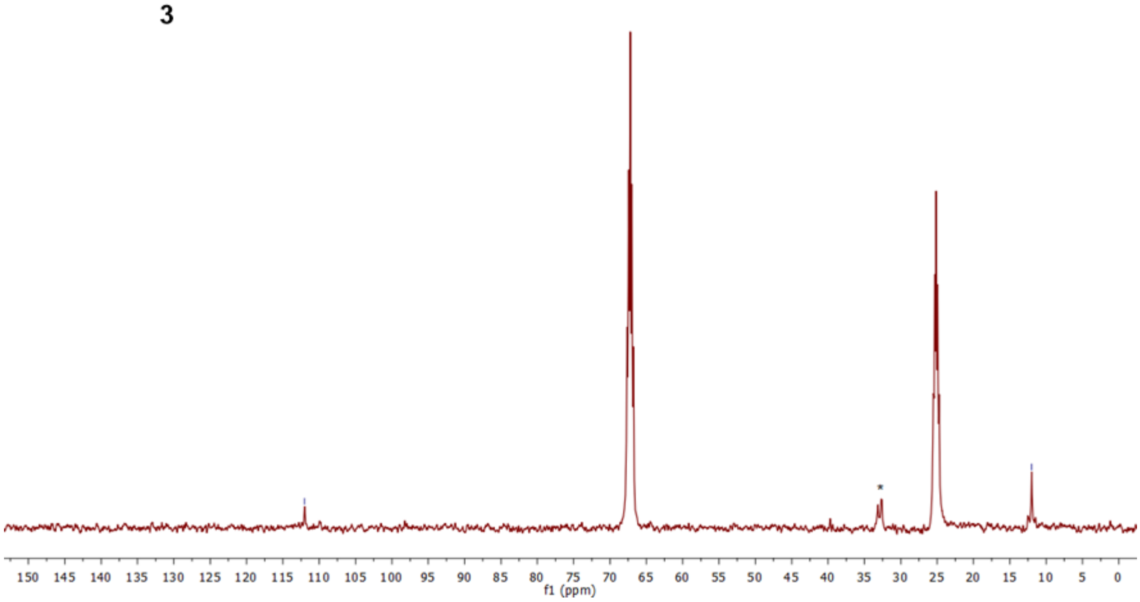

Figure S7. **^{13}C{^{1}H} NMR** (100 MHz, THF-d_8, 25 ºC).Compound **3**. * Cp*H

7. References

[1] Goel, R. G.; Ogini, W. O.; Srivastava, R. C. Preparation, Characterization and Some Reactions of Bis(tri-tert-butylphosphine)hydridoplatinum(II) Complexes. *Organometallics* **1982**, *1*, 819–824.

[2] Goel, R. G.; Srivasta, R. C. Preparation, Characterization, and Some reactions of Hydridobis(tri-tert-butyl)phosphineplatinum(II) Cation Containing Three-coordinate Platinum. *Can. J. Chem.* **1983**, *61*, 1352–1358.

[3] Sheldrick GM. SADABS, Program for Empirical Absorption Correction of Area Detector Data. Göttingen: University of Göttingen; **1996**.

[4] G. M. Sheldrick, SHELXTL, version 6.14. Program for solution and refinement of crystal structures, Universität Göttingen, Germany, **2000**.

[5] Gaussian 16, Revision C.01, Frisch, M. J.; Trucks, G. W.; Schlegel, H. B.; Scuseria, G. E.; Robb, M. A.; Cheeseman, J. R.; Scalmani, G.; Barone, V.; Petersson, G. A.; Nakatsuji, H.; Li, X.; Caricato, M.; Marenich, A. V.; Bloino, J.; Janesko, B. G.; Gomperts, R.; Mennucci, B.; Hratchian, H. P.; Ortiz, J. V.; Izmaylov, A. F.; Sonnenberg, J. L.; Williams-Young, D.; Ding, F.; Lipparini, F.; Egidi, F.; Goings, J.; Peng, B.; Petrone, A.; Henderson, T.; Ranasinghe, D.; Zakrzewski, V. G.; Gao, J.; Rega, N.; Zheng, G.; Liang, W.; Hada, M.; Ehara, M.; Toyota, K.; Fukuda, R.; Hasegawa, J.; Ishida, M.; Nakajima, T.; Honda, Y.; Kitao, O.; Nakai, H.; Vreven, T.; Throssell, K.; Montgomery, J. A., Jr.; Peralta, J. E.; Ogliaro, F.; Bearpark, M. J.; Heyd, J. J.; Brothers, E. N.; Kudin, K. N.; Staroverov, V. N.; Keith, T. A.; Kobayashi, R.; Normand, J.; Raghavachari, K.; Rendell, A. P.; Burant, J. C.; Iyengar, S. S.; Tomasi, J.; Cossi, M.; Millam, J. M.; Klene, M.; Adamo, C.; Cammi, R.; Ochterski, J. W.; Martin, R. L.; Morokuma, K.; Farkas, O.; Foresman, J. B.; Fox, D. J. Gaussian, Inc., Wallingford CT, **2016**.

[6] (a) Becke, A. D. Density-functional Exchange-energy Approximation with Correct Asymptotic Behavior. *Phys. Rev. A* **1988**, *38*, 3098–3100. (b) Perdew, J. P. Density-functional Approximation for the Correlation Energy of the Inhomogeneous Electron Gas. *Phys. Rev. B* **1986**, *33*, 8822–8824.

[7] Weigend, F.; Ahlrichs, R. Balanced Basis Sets of Split Valence, Triple Zeta Valence and Quadruple Zeta Valence Quality for H to Rn: Design and Assessment of Accuracy. *Phys. Chem. Chem. Phys*. **2005**, *7*, 3297–3305.

[8] Grimme, S.; Antony, J.; Ehrlich, S.; Krieg, H. A Consistent and Accurate *ab initio* Parametrization of Density Functional Dispersion Correction (DFT-D) for the 94 Elements H-Pu. *J. Chem. Phys*. **2010**, *132*, 154104.

[9] (a) Huzinaga, S.; Miguel, B. A Comparison of the Geometrical Sequence Formula and the Well-tempered Formulas for Generating GTO Basis Orbital Exponents *Chem. Phys. Lett*. **1990**, *175*, 289–291. (b) Huzinaga, S.; Klobukowski, M. Well-tempered Gaussian Basis Sets for the Calculation of Matrix Hartree—Fock Wavefunctions. *Chem. Phys. Lett*. **1993**, *212*, 260–264.

[10] Cabeza, J. A.; van der Maelen, J. F.; García-Granda, S. Topological Analysis of the Electron Density in the N-Heterocyclic Carbene Triruthenium Cluster [Ru_3(μ-H)$_2$(μ$_3$-MeImCH)(CO)$_9$] (Me_2Im = 1,3-dimethylimidazol-2-ylidene). *Organometallics* **2009**, *28*, 3666–3672 and references therein.

[11] Keith, T. A. AIMAll, **2010**, http://tkgristmill.com.

[12] For a recent review, see: von Hopffgarten, M.; Frenking, G. Energy Decomposition Analysis. *WIREs Comput. Mol. Sci*. **2012**, *2*, 43–62.

[13] ADF program: www.scm.com.

[14] Snijders, J. G.; Vernooijs, P.; Baerends, E. J. Roothaan-Hartree-Fock-Slater atomic wave functions: Single-zeta, Double-Zeta, and Extended Slater-type Basis sets for $_{87}$Fr-$_{103}$Lr. *At. Data. Nucl. Data Tables* **1982**, *26*, 483–509.

[15] Krijn, A.; Baerends, E. J.; Fit Functions in the HFS-Method, Internal Report (in Dutch), Vrije Universiteit Amsterdam, The Netherlands, **1984**.

[16] (a) van Lenthe, E.; Baerends, E. J.; Snijders, J. G. Relativistic Regular Two-component Hamiltonians. *J. Chem. Phys.* **1993**, *99*, 4597–4610. (b) van Lenthe, E.; Baerends, E. J.; Snijders, J. G. Relativistic Total Energy Using Regular Approximations. *J. Chem. Phys.* **1994**, *101*, 9783–9792. (c) van Lenthe, E.; Ehlers, A.; Baerends, E. J. Geometry Optimizations in the Zero Order Regular Approximation for Relativistic Effects. *J. Chem. Phys.* **1999**, *110*, 8943–8953.
